# Trimeric Structure of (+)-Pinoresinol-forming Dirigent Protein at 1.95 Å Resolution with Three Isolated Active Sites*

**DOI:** 10.1074/jbc.M114.611780

**Published:** 2014-11-19

**Authors:** Kye-Won Kim, Clyde A. Smith, Michael D. Daily, John R. Cort, Laurence B. Davin, Norman G. Lewis

**Affiliations:** From the ‡ Institute of Biological Chemistry, Washington State University, Pullman, Washington 99164-6340,; the § Stanford Synchrotron Radiation Lightsource, Menlo Park, California 94025, and; the ¶ Fundamental and Computational Sciences Directorate, Pacific Northwest National Laboratory, Richland, Washington 99354

**Keywords:** Crystallography, Homology Modeling, Molecular Docking, Protein Crystallization, Secondary Metabolism, Dirigent Proteins, Phenoxy Radical Radical Coupling

## Abstract

Control over phenoxy radical-radical coupling reactions *in vivo* in vascular plants was enigmatic until our discovery of dirigent proteins (DPs, from the Latin *dirigere*, to guide or align). The first three-dimensional structure of a DP ((+)-pinoresinol-forming DP, 1.95 Å resolution, rhombohedral space group H32)) is reported herein. It has a tightly packed trimeric structure with an eight-stranded β-barrel topology for each DP monomer. Each putative substrate binding and orientation coupling site is located on the trimer surface but too far apart for intermolecular coupling between sites. It is proposed that each site enables stereoselective coupling (using either two coniferyl alcohol radicals or a radical and a monolignol). Interestingly, there are six differentially conserved residues in DPs affording either the (+)- or (−)-antipodes in the vicinity of the putative binding site and region known to control stereoselectivity. DPs are involved in lignan biosynthesis, whereas dirigent domains/sites have been implicated in lignin deposition.

## Teaser

**Background:** Dirigent protein (DP) discovery gave new paradigm for monolignol-derived coupling *in planta.*

**Results:** (+)-Pinoresinol-forming DP (PsDRR206) three-dimensional structure was obtained at 1.95 Å resolution.

**Conclusion:** The tightly packed trimeric DP has three putative substrate binding sites spatially far apart, suggesting that each site involves monomer coupling directly.

**Significance:** New insights into monolignol radical-radical coupling *in planta*.

## Introduction

In terrestrial vascular plants, monolignol coupling affords the lignans (1), as well as the polymeric structural lignins (2), with the latter being the most abundant plant biopolymers next to cellulose. The former are, however, a very diverse class of vascular plant natural products, typically dimers and/or higher oligomers, whose physiological roles *in planta* are considered mainly involved in plant defense, particularly in leaf, (heart)wood, knot, and seed coat tissues. Many lignans are optically active and can have diverse coupling modes, although the 8–8′ interunit (dimer) linkage is perhaps the most commonly encountered (1). Some are also medicinally important, such as podophyllotoxin, or its semi-synthetic derivatives, etoposide, teniposide, and Etopophos® widely used in cancer therapies (1).

**FIGURE 1.**
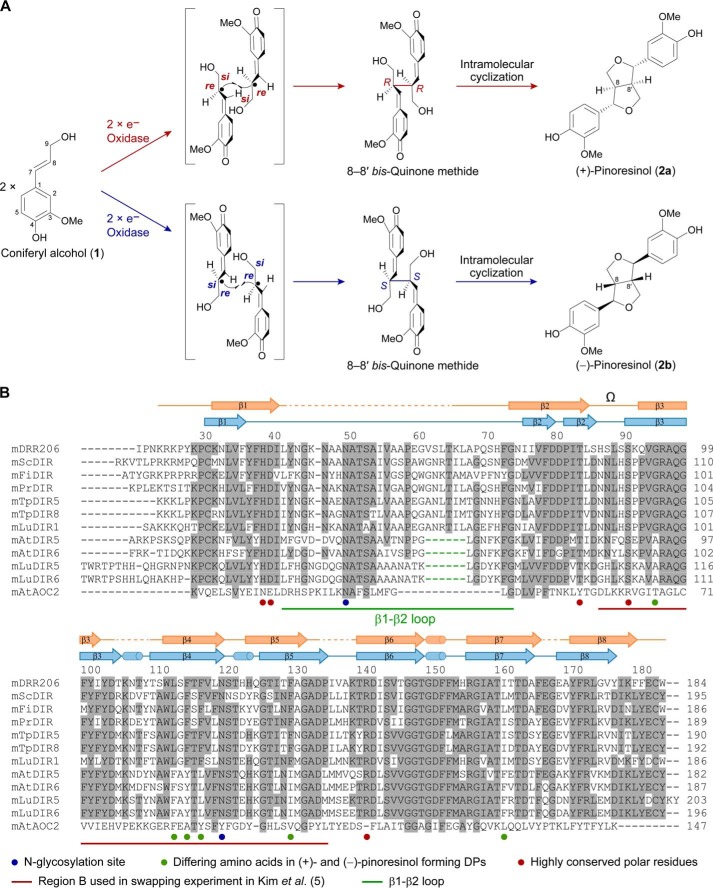
**Pinoresinol-forming dirigent proteins.** *A*, proposed biosynthesis mechanism to afford (+)- or (−)-pinoresinols (**2a** or **2b**) in DP-guided stereoselective couplings with coniferyl alcohol (**1**) as substrate. *B*, amino acid sequence alignments of mature (+)- and (−)-pinoresinol-forming DPs with their structural homologue AtAOC2. Secondary structures of PsDRR206 (*orange*) and AtAOC2 (*blue*) are also drawn. Loop residues in the PsDRR206 structure absent in the (−)-pinoresinol-forming DPs are indicated with *green dashed lines* in the sequences.

**FIGURE 2.**
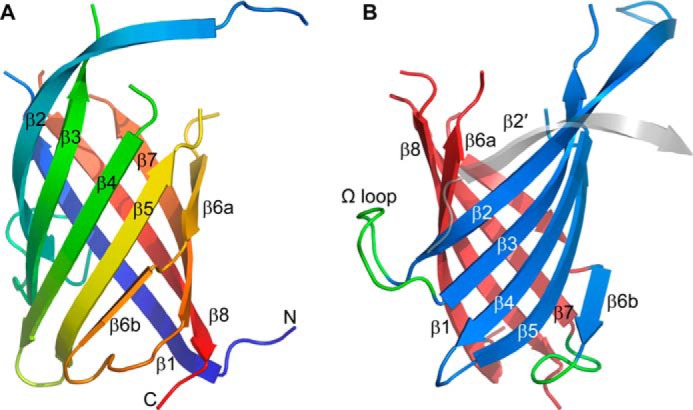
**Structure of the PsDRR206 monomer.** *A*, ribbon representation of the eight-stranded β-barrel rainbow-colored from the N terminus (*blue*) to the C terminus (*red*), showing the secondary structure labeling. *B*, the PsDRR206 monomer rotated 90° about a vertical axis and colored to show the two component β-sheets in *blue* (β-sheet 1) and *red* (β-sheet 2). The location of the domain-swapped strand β2′ from a neighboring PsDRR206 molecule making up the trimer is shown as a *transparent gray strand*. The two long loops between strands β2/β3 and β6/β7 are colored *green*.

The entry point into presumed phenoxy radical-radical coupling to the 8–8′ lignan class involves one electron oxidation of the monolignol, *e.g.* coniferyl alcohol (Fig. 1*A*, **1**), which in the presence of a specific type of dirigent protein (DP; from the Latin *dirigere*, to guide or to align)^2^ (3), can undergo distinct forms of stereoselective coupling to afford either the (+)- or the (−)-antipode of pinoresinol (Fig. 1*A*, **2a** or **2b**) (1, 3–6). Mechanistically, one electron oxidation (*e.g.* catalyzed by laccases, peroxidases) is envisaged to generate the corresponding coniferyl alcohol (**1**)-derived free radical intermediate (CA^•^), which then binds to the DPs. Bound substrate orientation subsequently occurs in such a way as to enable 8–8′ coupling at the *si-si* face with concomitant intramolecular cyclization to afford (+)-pinoresinol (Fig. 1*A*, **2a**), such as in *Forsythia intermedia* (3), *Thuja plicata* (4), *Schizandra chinensis* (5), or pea (*Pisum sativum*) (7), or alternatively *re-re* coupling to give the (−)-antipode (Fig. 1*A*, **2b**) as in *Arabidopsis* (1, 5, 6) and flax (*Linum usitatissimum*).^3^ Of these, the (+)-pinoresinol-forming DP in pea is annotated as PsDRR206 (9), and the (−)-pinoresinol-forming DP in *Arabidopsis thaliana* is named AtDIR6 (5).

In this investigation, determination of the x-ray crystal structure of the (+)-pinoresinol-forming DP PsDRR206 is reported. These results are discussed with regard to previously proposed mechanisms for DP-mediated CA^•^ coupling (10) and a published homology model of AtDIR6 (11), as well as existing x-ray structures of allene oxide cyclase (AOC) (12, 13). AOC is a plant enzyme that forms 12-oxo-phytodienoic acid, an intermediate in jasmonic acid biosynthesis, from a reactive allene oxide precursor, 12,13-epoxyoctadeca-9,11,15-trienoate (14). AOC shares distant sequence similarity to DPs.

## EXPERIMENTAL PROCEDURES

### 

#### 

##### Cloning, Expression, and Purification of PsDRR206

The PsDRR206 gene (GenBank^TM^ accession number U11716) was isolated from pea (*P. sativum* cv. Alcan) genomic DNA using PCR amplification with gene specific primers (DRR206 forward, ATGGGTTCCAAACTTCTAGTACTA, and DRR206 reverse, TTACCAACACTCAAAGAACTTGAT). Vector construction and transformation were carried out using biolistic bombardment technology as described by Seneviratne *et al.* (7), as was expression and purification of the (+)-pinoresinol-forming PsDRR206 recombinant DP in tomato cell culture.

##### Crystallization and X-ray Data Collection

Initial crystallization conditions for the PsDRR206 protein were obtained using the microbatch under oil method employing 1536-well microassay plate high throughput screening (15) at the Hauptman Woodward Institute (Buffalo, NY). Four conditions from preliminary screening produced microcrystals: condition 265, 0.1 m (NH_4_)_2_SO_4_, 0.1 m CAPS pH 10, PEG8000 20% (v/v); condition 1006, 0.1 m NH_4_Br, 0.1 m Tris, pH 8, PEG 400 40% (v/v); condition 1077, 5% 1-butyl-2,3-dimethylimidazolium tetrafluoroborate (w/v), 0.09 m Bis-Tris propane, pH 7.8, and 27% PEG 3350; and condition 1474, 0.1 m NaCl, 0.1 m NaOAc, pH 5.0, PEG 400 80% (w/v). Initial “hits” were scaled up in-house as hanging drop vapor diffusion methods on VDX 24-well plates (Hampton Research, CA) incubated at 22 °C with a drop size of 3 μl consisting of equal volumes of protein at a concentration of 4–5 mg/ml in 40 mm MES, pH 5, 20 mm Na_2_SO_4_, 20% glycerol, and reservoir solution. Each drop was equilibrated against a 500-μl reservoir and monitored periodically. Crystallization conditions were further optimized using grid screening. Diffraction quality crystals were obtained from the final optimized crystallization condition 1077, comprising 4% 1-butyl-2,3-dimethylimidazolium tetrafluoroborate (w/v), 0.1 m Bis-Tris propane, pH 7.8, and 35% PEG 3350. Crystals appeared within 5–10 days and grew to the size of 170 × 170 × 150 μm within 10 weeks. The PsDRR206 crystals were subsequently flash cooled in a cryoprotectant crystallization buffer, stored in cryovials, and shipped to the Stanford Synchrotron Radiation Lightsource for data collection.

Native PsDRR206 crystals, diffracting to ∼1.95 Å resolution, belong to the rhombohedral space group H32 (see Table 1). A complete data set comprising 600 images with a rotation angle of 0.2° was collected from a single PsDRR206 crystal on Stanford Synchrotron Radiation Lightsource Beamline BL12-2 using x-rays at 12,658 eV (0.98093 Å) and a PILATUS 6 m PAD detector running in the shutterless mode. The data were processed with XDS (16) and scaled with SCALA from the CCP4 suite of programs (17). The Matthews coefficient (18) assuming two molecules in the asymmetric unit was 2.0 Å^3^/Da (39% solvent content). The final data collection statistics are given in Table 1 (19).

##### Data Processing, Structure Determination, and Refinement

PsDRR206 structure was solved by molecular replacement using the homology model generated for the (−)-pinoresinol-forming DP AtDIR6 from *A. thaliana* (PM0078038 (11)), which was derived from an x-ray structure of *A. thaliana* AOC2 (AtAOC2; PDB code 2BRJ (12)). The sequences of PsDRR206 and AtDIR6 were aligned and the AtDIR6 model was converted into a pseudo-DRR206 model using the program CHAINSAW (20) from the CCP4 suite (17). Identical residues in the two sequences were retained, and those that differed were truncated at the Cβ atom. A weak molecular replacement solution, with a weighted *R* factor of 0.61, and a score of 0.42 was obtained with the program MOLREP (21). This solution was refined for 15 cycles using REFMAC (22) giving crystallographic *R* factor and *R*_free_ values of 0.48 and 0.54, respectively, and 2*F*_o_ − *F*_c_ and *F*_o_ − *F*_c_ electron density maps were calculated. Inspection of the electron density showed that the molecular replacement solution was correct, with side chains truncated by CHAINSAW clearly visible in the 2*F*_o_ − *F*_c_ and *F*_o_ − *F*_c_ maps. Refinement of PsDRR206 structure was completed with the PHENIX suite of programs (23) and manual building of the model using the molecular graphics program COOT (24). Water molecules were added at structurally and chemically relevant positions, and the atomic displacement parameters for all atoms in the structure were refined isotropically. The final refinement statistics are given in Table 1.

Atomic coordinates and structure factors for PsDRR206 were deposited to the Protein Data Bank (25) with PDB code 4REV. Superpositions were performed using the SSM procedure (26) as implemented in COOT (24) and the program LSQKAB in the CCP4 suite (17). The figures were generated using PyMOL (27). The surface topography was analyzed with CASTp (28) using a probe radius of 1.4 Å. Conserved residues were mapped onto the structure using ConSurf (29).

##### Computational Docking Experiments

PsDRR206 and two ligands were prepared in AutoDockTools4 (30) with default bonded and nonbonded parameters. One ligand approximated the coniferyl alcohol quinone methide radical (CA^•^), with C7 and C8 set to be trivalent, trigonal planar carbon atoms coplanar with the ring carbon and quinone oxygen atoms. A second ligand was the 8–8′ linked *bis*-quinone methide. Both *syn* and *anti* isomers, referring to the relationship between C8 and the methoxyl group, were used in the docking experiments, and rotation about the C7-C8 bond was not allowed in CA^•^, reflecting its partial double bond character in the resonance hybrid. Flexibility was allowed for the side chains of residues Asp^40^, Phe^79^, Tyr^101^, Ser^111^, Leu^113^, Ile^161^, Thr^163^, and Leu^174^. The grid box was centered upon the ligand with sufficient size to cover the ligand, and all flexible residues and a grid spacing of 0.375 Å. Autodock Vina (31) was used to perform the docking calculations.

##### Homology Modeling

Homology models of AtDIR6 were built in SWISS-MODEL (32).

## RESULTS

Fungally challenged pea (*P. sativum*) pods have an overall non-host disease response that includes induction of a gene called *PsDRR206* (*Disease Resistance Response 206*), whose protein biochemical function until recently (7) was unknown (33–35). PsDRR206 shares 54% sequence identity with AtDIR6 over the extent of the mature polypeptide. For its study *in vitro*, recombinant PsDRR206 was constitutively expressed using the strong CaMV ^35^S promoter of pART17 (5). Large scale (3 liters) suspension culture of transformed tomato (*Solanum peruvianum*) cells was obtained by gradually increasing the culture volume (starter volume, 40 ml) with fresh liquid medium for 4 weeks at weekly subculturing intervals. Proteins were isolated using a series of strong cationic column chromatographic steps (7) with ∼400 μg of PsDRR206 protein obtained from the 3-liter culture.

Detailed biochemical characterization next established it to be a (+)-pinoresinol-forming DP (7), and a homologue to our much earlier discovery of this protein type and its physiological function from *Forsythia* species (3). Thus we have now discovered DPs producing both pinoresinol antipodes, with the recent discovery of a (−)-pinoresinol-forming DP in *Arabidopsis* (AtDIR6) (1, 5). Interestingly, the function of AtDIR6 was also reported independently by others (6). Of the various DPs under investigation thus far, however, crystallization conditions have only been identified for PsDRR206, the focus of this investigation.

### 

#### 

##### Overall Structure

PsDRR206 structure was solved by molecular replacement and refined at 1.95 Å resolution (Table 1). The asymmetric unit contains two independent molecules related by a noncrystallographic 2-fold rotation axis and connected by a single salt bridge between Glu^169^ and Arg^173^. Molecule A comprises residues 28–43, 65–105, 110–135, 138–166, and 169–184, whereas molecule B contains residues 28–42, 66–104, 109–135, 138–166, and 168–184 (see PDB code 4REV for details). Superposition of the two independent molecules gives a root mean square deviation (RMSD) of 0.4 Å for 124 matching Cα atoms. The structure of the PsDRR206 monomer (Fig. 2*A*) can be best described as an eight-stranded antiparallel β-meander forming a β-barrel. The displacement (shear number) at the suture between β1 and β8, caused by twisting of the β-sheet, is ten residues. The gaps in the structure correspond to the seven N-terminal residues and loops between strands β1–β2, β3–β4, β5–β6a, and β7–β8. In all of these gaps, the electron density was either very weak or missing completely, such that the residues in the loops could not be fitted. Interestingly, the four missing loops are all at one end of the β-barrel, designated here as the top of the molecule (Fig. 2, *A* and *B*). *N*-Linked glycans assumed to be present at Asn^50^ and Asn^120^ in PsDRR206 based on their occurrence at the equivalent residues in AtDIR6 (11) are also missing in the electron density map.

**TABLE 1:** **Data collection and refinement statistics for PsDRR206**

**Data collection**	
Space group	H32
Maximum resolution (*d*_min_) (Å)	1.95
Unit cell dimensions (Å)	*a* = *b* = 88.4, *c* = 196.8
Observed reflections	142,020
Unique reflections to *d*_min_	21,935
*R*_merge_ (%)*^a^*	3.7 (66.1)*^b^*
*I*/σ	21.9 (2.6)
Completeness (%)	99.6 (98.1)
CC½*^c^*	100.0 (85.7)
Multiplicity	6.5 (5.4)
Wilson B (Å^2^)	39.6

**Refinement**	
Resolution range (Å)	38.5–1.95
*R* factor/*R*_free_ (%)*^d^*	20.1/24.8
*R*_all_ (%)*^e^*	
Total atoms	
Protein	1030/1012
Solvent	50
B factors	
Protein chain (A/B) (Å^2^)	59.1/59.4
Solvent (Å^2^)	57.5
RMSD from ideality	
Bonds (Å)	0.009
1–3 distances (Å)	1.43
Ramachandran plot	
Residues in preferred regions (%)*^f^*	98.2

*^a^ R*_merge_ = Σ|*I* − <*I*>|/Σ*I* × 100, where *I* = the observed intensity, and <*I*> is the mean intensity.

*^b^* Numbers in parentheses relate to the highest resolution shell, 2.00–1.95 Å.

*^c^* Percentage of correlation between intensities from random half-sets calculated by XDS (16).

*^d^ r* = Σ||*F*_o_| − *k*|*F*_c_ ||/Σ|*F*_o_ | × 100. *R*_free_ was calculated with 5% of the reflections.

*^e^* Final *R* factor calculated with all data using no sigma cutoff.

*^f^* As defined in MOLPROBITY (19).

Alternatively, the β-barrel can be viewed as two highly curved anti-parallel sheets formed by strands β2, β3, β4, and β5 (β-sheet 1) and by strands β6, β7, β8, and β1 (β-sheet 2) (Fig. 2*B*), respectively. Moreover, strand β6 is divided into two shorter strands such that the first part of the strand (β6a) forms the outer strand of β-sheet 2, whereas the C-terminal end (β6b) adds a fifth strand to β-sheet 1. The bifurcation of β6 occurs at a β-bulge at residues Val^145^-Thr^146^; a similar bulge is present at the equivalent position in AOC.

**FIGURE 3.**
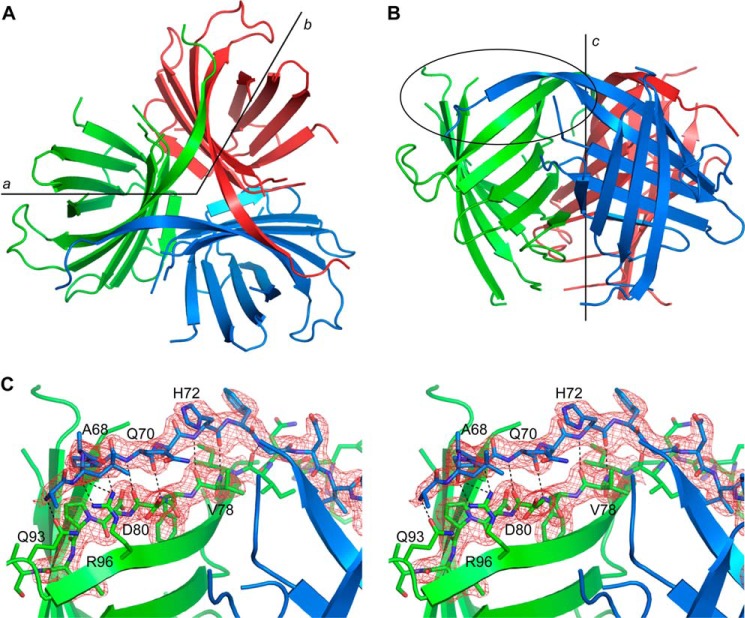
**Structure of the PsDRR206 trimer.** *A*, the trimer viewed down the 3-fold *c*-axis of the H32 space group. The *a*- and *b*-axes are indicated. *B*, the trimer rotated 90° about the *a*-axis. One of the extended β2 strands (*blue*) traverses the trimer interface into neighboring monomer (*green*), and this region is highlighted by the *black oval. C*, final 2*F*_o_ − *F*_c_ electron density, contoured at 1.0 σ, in the vicinity of the domain-swapped β2 strand from the *blue* monomer. This extended β2 strand makes a typical anti-parallel hydrogen bonding network with the β2 strand from the *green* monomer, along with interactions with two residues from the omega loop between strands β2 and β3 (Gln^93^ and Arg^96^). Some residues in the *blue* and *green* β2 strands are indicated.

**FIGURE 4.**
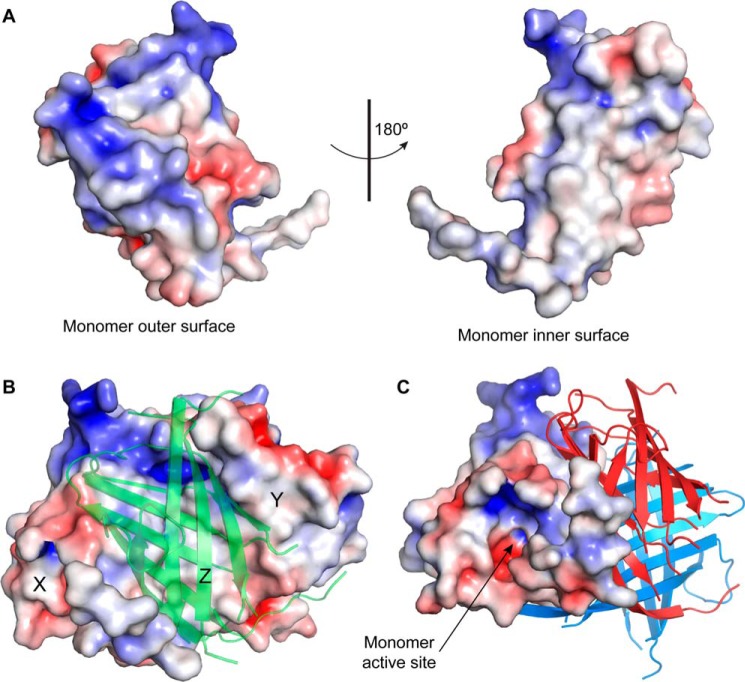
**Surface representation of PsDRR206.** *A*, the outer (solvent exposed, *left panel*) and the inner (buried in trimer formation, *right panel*) electrostatic surfaces of the monomer. The surface potentials range from −4 kT/e (*red*) to +4 kT/e (*blue*). *B*, the electrostatic surface of two of the monomers, X and Y, with the third monomer (Z) indicated as a semi-transparent green ribbon. The inner surface of the Z monomer is represented by the *right panel* in *A*, rotated 180° about a vertical axis relative to the orientation of the *green ribbon. C*, the electrostatic surface of one of the monomers orientated to view down into the putative active site. The other two monomers making up the trimer are colored *red* and *blue*, and the orientation of the trimer is a 60° rotation about the *a*-axis relative to *A*.

Analysis of the crystal symmetry shows that PsDRR206 forms a tightly packed trimer, with the trimer axis being orientated along the 3-fold axis of the H32 space group (Fig. 3). Nevertheless, formation of the trimer buries almost 2500 Å^2^ of surface per monomer and over 30% of the total surface of each monomer. Calculation of the electrostatic surface of the PsDRR206 monomer shows that the outer surface is hydrophilic (*left side* of Fig. 4*A*), whereas the inner surface involved in trimer interface formation is predominantly hydrophobic (Fig. 4*A, right side*, and *B*). Along with a large number of hydrophobic contacts at the trimer interface, the monomers are also held together by six hydrogen bonding interactions (three for each monomer-monomer pair), and two electrostatic interactions (not shown). Inspection of the trimer shows that the loop between the first two strands is involved in a domain-swapping event (Fig. 3*B*), wherein the loop does not simply reverse direction and become the second β-strand in the same monomer. Instead, it traverses the interface into the neighboring monomer, such that the N-terminal half of the first strand β2 is inserted into the second monomer next to its strand β2 (Fig. 3, *A* and *B*), adding a sixth strand to β-sheet 1. The electron density in this region clearly shows the N-terminal extension of this strand (Fig. 3*C*). This extension of strand β2 provides an additional nine hydrogen bonds, which include seven main chain interactions with strand β2 from the second monomer and two interactions with side chain atoms from the β2-β3 loop (Fig. 3*C*).

**FIGURE 5.**
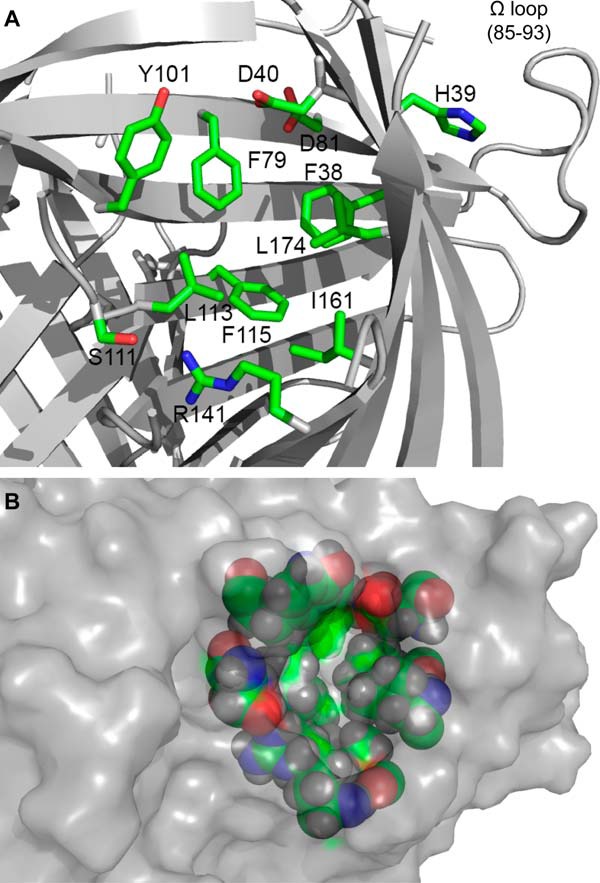
**Residues lining the interior of the active site pocket.** *A*, several of these residues are conserved, although in only one or the other but not both of the groups of known (+)- or (−)-pinoresinol-forming DPs as indicated in Fig. 1*B*, suggesting a role in determining substrate orientation. The omega loop is also labeled to indicate proximity to the back side of the active site. *B*, surface rendering of the active site pocket and surrounding area from the same perspective as *A*, with residues shown as *sticks* in *A* now shown as *colored spheres* visible under the transparent surface.

At the top of each barrel and slightly toward the outside of the trimer is a substantial pocket lined mostly with hydrophobic residues (Figs. 4*C* and 5, *A* and *B*). Several conserved residues cluster around this pocket including strictly conserved charged polar residues Asp^40^ and Arg^141^ (Fig. 5*A*). This narrow pocket is approximately 12 Å deep, 12 Å wide, and 5 Å across. The solvent excluded molecular surface area (Connolly surface) of the pocket is 339 Å^2^, and its volume is 463 Å^3^.

Adjacent to Asp^40^ and on the opposite side of the β-sheet is found His^39^, one of three conserved residues in an unusual cluster of highly conserved residues that also includes Thr^84^ and Ser^91^. The latter two residues occur in an omega loop between strands β2 and β3 that folds back upon the exterior of the barrel (Figs. 2*B* and 5*A*). An omega loop is a nonregular secondary structure element characterized by a loop of varying structure with its N and C termini located on adjacent secondary structure elements, typically β-strands, close together in space (36).

##### Comparison of PsDRR206 to AOC

A Dali search of the PDB (37) identified several β-barrel proteins as being closely related to PsDRR206, including AOC (the first nine hits with Z scores ranging from 12.1 to 11.5), followed by five proteins of unknown function (PDB codes 2Q03, 3G7G, 2OOJ, 3C5O, and 4PUX) with Z scores between 10.5 and 7.8. Unlike AOC, these latter proteins do not form trimeric structures. The Dali search also identified numerous structures from the avidin/streptavidin fold family, but all with Z scores less than 7.0. Like AOC, these are eight-stranded β-barrel proteins with shear number 10; however, they meander in the opposite direction around the barrel.

**FIGURE 6.**
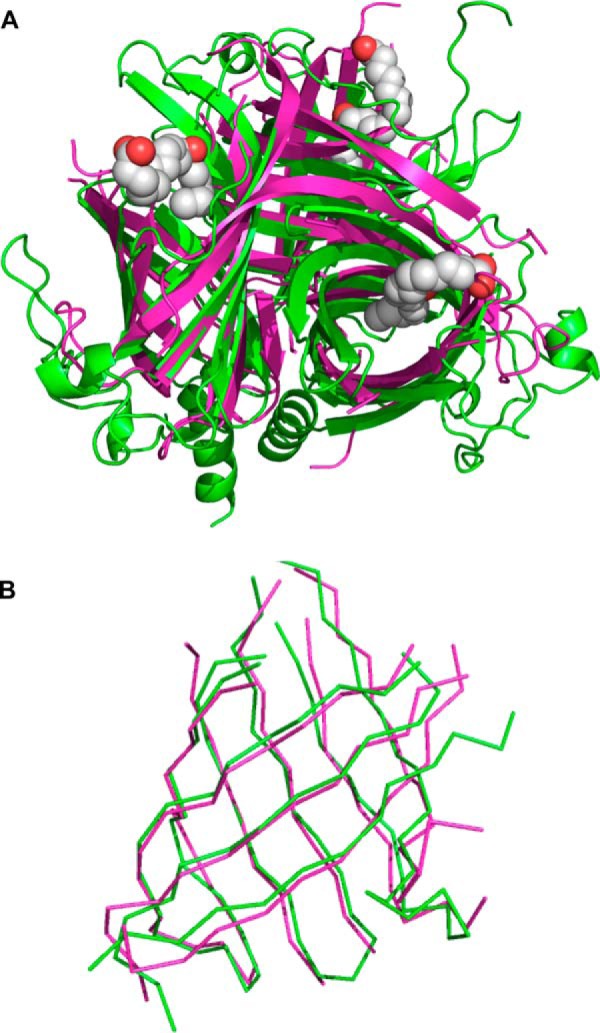
**PsDRR206 comparison with AOC.** *A*, PsDRR206 (*magenta*) superposition with *P. patens* AOC (4H69, *chartreuse*; with ligand, *spheres*), with RMSD ∼2.1–2.2 Å over 102 residues. *B*, aligned (pairwise Dali) residues only, Cα trace, for one monomer. All 102 residues are in the core β-barrel.

A structure-based sequence alignment of PsDRR206 and the highest scoring Dali hit, *Physcomitrella patens* AOC (PpAOC2; PDB code 4H69) (13) with bound inhibitor, identified 109 residues within spans of 151 and 164 residues in the PsDRR206 and AOC crystal structures, respectively, that could be aligned with 2.2 Å RMSD for the backbone atoms (Fig. 6, *A* and *B*). Similar alignments can also be generated with AtAOC2 structures (12). Most of the aligned residues constitute the common core barrel structure of both proteins, whereas the unaligned portions are in loops linking β-strands. Many of these loops also have substantial insertions and deletions, relative to their counterparts in the other structure. However, the prominent pocket or cleft on each PsDRR206 monomer aligns well with its counterpart in the AOC structure. The shapes of the PsDRR206 and AOC pockets are somewhat similar (Fig. 6*A*), and their volumes are also quite similar. The size and length of AOC ligands such as vernolic acid (PDB code 2DIO) are comparable with pinoresinol (**2**).

##### Computational Docking Studies

**FIGURE 7.**
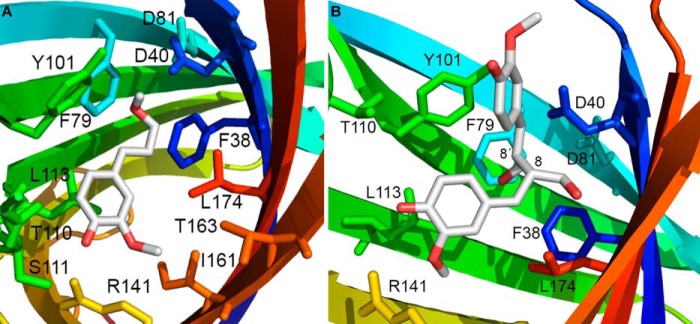
**Docked models of substrates en route to (+)-pinoresinol (2a), in the putative active site of PsDRR206.** *A,* coniferyl alcohol radical (CA^•^) in the *syn* configuration bound such that the *re* face is exposed, the orientation that would lead to coupling with the *re* face of another CA^•^ to give the *R*,*R*-stereoisomer of 8–8′ *bis*-quinone methide, the putative initial product from coupling of two coniferyl alcohol radicals as shown in *B*. In this particular model of the *bis*-quinone methide, the 3 and 3′-methoxy groups are *anti* with respect to C8 and C8′; models with *syn* regiochemistry were also docked. The torsion angle about the 8–8′ bond was restricted to values in which cyclization to form the furan rings of pinoresinol (**2**) would produce the correct configuration at C7 and C7′. PsDRR206 side chains allowed to be flexible in the docking simulation are labeled and shown as *sticks*.

Because the putative substrate for PsDRR206 is a reactive radical species, experimental investigation of the protein-substrate complex is not straightforward. Accordingly, computational docking studies were conducted using the PsDRR206 x-ray structure and models of both the coniferyl alcohol quinone methide radical (CA^•^) and the 8–8′ *bis*-quinone methide, with the latter being the putative intermediate in pinoresinol (**2**) formation following (CA^•^) radical coupling prior to cyclization of the furan rings (Fig. 1*A*). These docking studies suggest that the binding site can accommodate at least one CA^•^ (Fig. 7*A*) and that its dimensions are appropriate for the long axis to span the distance from Arg^141^ to Asp^40^, with the quinone oxygen and the 9-OH potentially interacting favorably with these charged residues.

## DISCUSSION

The study herein obtained the (+)-pinoresinol-forming DP (PsDRR206) in crystalline form, and at 1.95 Å resolution. In this regard, determination of the PsDRR206 three-dimensional structure, and its analysis suggests that the corresponding trimer has three identical substrate binding sites that are spatially distributed so that intermolecular coupling apparently cannot occur between two distinct substrate binding sites in the trimer model. Furthermore, this trimer observation is in agreement with our earlier studies (3), where the *Forsythia* DP was considered as a ∼78-kDa trimer based on gel filtration analyses, in contrast to subsequent mass spectrometric data obtained, which had suggested a dimeric protein (38).

As indicated above, we also established that PsDRR206 is an eight-stranded β-barrel with the same β-meander topology and remarkably similar trimeric quaternary structure as AOC. Together with the previously mentioned low (∼17% identity), but likely meaningful, sequence similarity between DP and AOC protein families, this suggests a possible common ancestor for these functionally distinct proteins.

Although there are striking structural similarities between PsDRR206 and AOC, it remains to be seen whether there are mechanistic similarities as well. Studies of AOC with and without a bound inhibitor suggested that conformational changes in the binding pocket upon formation of a tight enzyme-substrate complex precede the catalytic cycle and that steric restrictions in the active site determine the stereoselectivity of the cyclization reaction (12, 13). Both of these characteristics could be seen to be important in a protein guiding stereoselective phenoxy radical coupling. Conceivably, the trimeric β-barrel DP/AOC scaffold is well suited for these roles.

### 

#### 

##### DP Surface Features and Patterns of Conserved Residues

**FIGURE 8.**
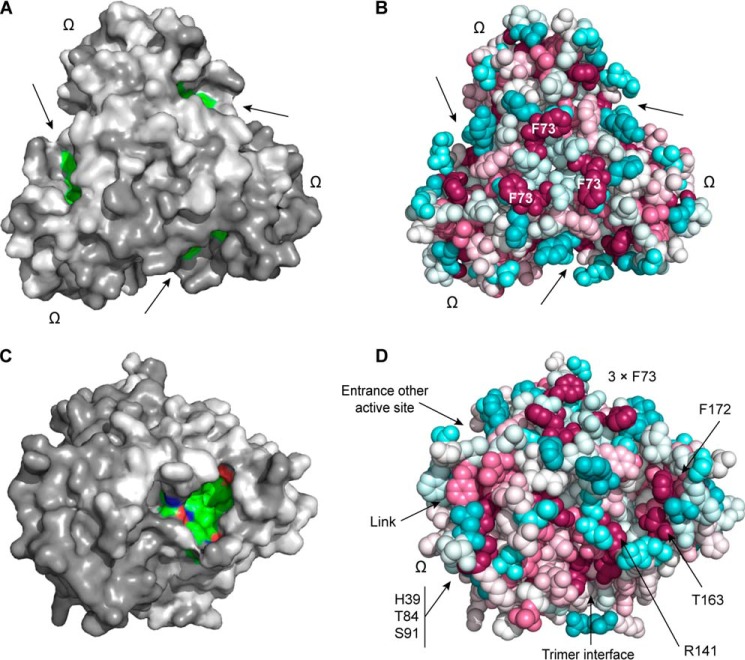
**Surface features and conserved residues.** *A*, top view of accessible surface showing that putative active site pockets (entrance indicated by *arrows*, residues colored by atom type) are isolated from each other in the trimer. The omega loop is also indicated. *B*, top view (same as in *A*) generated with ConSurf (29) showing conserved residue Phe^73^ in relation to the active site and omega loop. Conserved residues are shown in *magenta*. The sequence alignment input to ConSurf contained PsDRR206 and 16 dirigent proteins and their homologues from *Arabidopsis. C*, view directly into the active site, at ∼45° away from the top view shown in *A* and *B. D*, same view as in *C*, but with residues colored according to ConSurf. The network of conserved residues formed by the omega loop and by residues on the back side of the active site pocket is visible.

We hypothesize that the prominent outward facing pocket near the top of each barrel is the substrate binding (active) site located within the internal cavity of the barrel, based upon comparison with the structures and similar dimensions of *P. patens* AOC1 and AOC2 with bound substrate analog (PDB codes 4H69 and 4H6B (13)). We further propose that this is the binding site for one or possibly two coniferyl alcohol phenoxy radicals (CA^•^) in PsDRR206. Conserved residues Thr^163^ and Phe^172^ line the edge of the putative binding pocket (Fig. 8, *A–D*) (29), and disordered loop residues for which electron density is missing occur around the edge of the pocket. Two polar residues, Asp^40^ and Arg^141^, conserved in all pinoresinol-forming DPs but not AOCs, are also located at either end of the pocket (Figs. 5*A* and 7*A*). The span between them is ∼12–14 Å, suitable for a single *trans* coniferyl alcohol molecule or quinone methide radical, whose lengths are ∼8–10 Å. Residues in the interior of the pocket are less conserved, however, possibly because they help determine substrate specificity.

In PsDRR206, but not AOC, an omega loop consisting of approximately eight residues (Leu^85^–Lys^92^) occurs at the bottom of the trimer between β2 and β3 and folds back upon the outside of the barrel (Fig. 5*A*). A cluster of three highly conserved polar residues (His^39^, Thr^84^, and Ser^91^) also occur (Fig. 8*D*) where this loop contacts the exterior surface of the barrel. These three residues are conserved in all characterized (+)- and (−)-pinoresinol-forming DPs (Fig. 1*B*), whereas they are not conserved in AOCs. The cluster adjoins the active site pocket, and indeed His^39^ neighbors conserved residue Asp^40^ in the putative active site region, although on the opposite side of the strand in the β-barrel (Fig. 5*A*). This proximity of conserved residues thus provisionally suggests the omega loop may be the site of an interaction that modulates either substrate orientation or binding in the active site. As indicated above, because the proposed pockets point outwards relative to the trimer axis, a substrate in one pocket is unlikely to be able to couple with those in other pockets elsewhere in the trimer.

Another notable residue conserved in DPs, although not in AOCs, is Phe^73^ (Tyr in FiDIR and some other DPs), which lies near its symmetry mates on the exterior surface of the barrel at its topmost point and near the rim of the putative active site. This positioning suggests it may serve as a hydrophobic anchor point (Figs. 5 and 8 (*A* and *B*)). Its conservation and location on the surface suggest it is maintained there by selective pressure. Because they do not appear likely to be necessary for stabilization of the structure, these conserved surface-exposed hydrophobic side chains might instead indicate interaction site(s) for an oxidase, other dirigent proteins, or perhaps even a surface such as that of an actively growing lignin polymer. Several other conserved residues in DPs are also found buried in the trimer interface, supporting the idea that the trimer is the functional form of this and probably other DPs.

##### Comparison of Amino Acid Sequences for DPs with Opposite Stereoselectivities and Homology Models of AtDIR6

In our earlier work (5) and that of others (11), homology models of ScDIR and AtDIR6, (+)- and (−)-pinoresinol-forming DPs, respectively, were made using the very distantly related AOC structures as potential templates. The x-ray structure of PsDRR206, however, now allows construction of accurate homology models of AtDIR6 (∼50% amino acid identity to PsDRR206).

Modeling of missing loop residues permits consideration of loop regions surrounding the putative active site where weak or missing electron density prevented fitting of the structure. All of the known (+)-pinoresinol-forming DPs have six more residues in the longest of these loops, between strands β1 and β2 (Leu42 to Gly74 of PsDRR206), than the (−)-pinoresinol DPs. This difference is conserved in various plant species (Fig. 1*B*). This correlation with stereoselectivity may indicate a role for this loop in binding or orientation of the substrate, for example by acting as a “lid” that closes on the active site as substrates bind.

Furthermore, the precise residues essential for distinct stereoselectivities in PsDRR206 and AtDIR6 were of considerable interest, because previously this was only narrowed down to the Region B in the DPs (Fig. 1*B*) (5). That is, using a region swapping approach, we established regions in the DPs that affected different stereoselectivities resulting in formation of opposite antipodes of pinoresinol (**2**) (5).

Our recent characterization of pea (7) and flax (8) DPs, however, now expands the number of DPs of known function to eleven in seven different plant species, namely *F. intermedia*, *T. plicata*, *A. thaliana*, *S. chinensis*, *Piper regnellii*, *P. sativum*, and *L. usitatissimum*. Among them, seven DPs (FiDIR, TpDIR5, TpDIR8, ScDIR, PrDIR, PsDRR206, and LuDIR1) afford (+)-pinoresinol (**2a**), whereas four others (AtDIR5, AtDIR6, LuDIR5, and LuDIR6) generate (−)-pinoresinol (**2b**). Interestingly, flax has both (+)- and (−)-pinoresinol-forming DPs (LuDIR1 *versus* LuDIR5 and LuDIR6^3^, respectively).

Although previously we had noted 14 residues differentially conserved in (+)- and (−)-pinoresinol-forming DPs (5), the recent identification of pea and flax DPs enabled us to narrow down candidate residues further to six. These now include Gly^95^, Leu^113^, Phe^115^, Phe^117^, Phe^130^, and Ile/Leu^161^ in the (+)-pinoresinol-forming DPs (numbering is based on PsDRR206), as well as Ala^98^, Phe^116^, Tyr^118^, Leu^120^, Ile^133^, and Phe^164^ in the (−)-pinoresinol-forming DPs; the numbering is based on AtDIR6 (Fig. 1*B*, with residues marked with green dots). Interestingly, except for Ile^161^ of PsDRR206 (and Phe^164^ in the (−)-pinoresinol-forming DP), all of these residues are located in Region-B (Lys^90^–Leu^138^ of AtDIR6) that was previously found responsible for altered stereoselectivity by region swapping (5). Furthermore, residues Leu^113^ and Phe^115^ of PsDRR206 (Phe^116^ and Tyr^118^ of AtDIR6) are located within the putative active site region (Fig. 5, *A* and *B*), with both residues being on the β4 strand and having their side chains positioned at distances less than 10 Å to the conserved polar residues Asp^40^ and Arg^141^; the latter provisionally appears to be positioned in a way that could determine whether the *si* or *re* face of bound CA^•^ is exposed and available for coupling. Accordingly, in the future, site-directed mutagenesis will examine whether one or more of the six residues determine distinct stereoselectivities.

##### Homology Modeling of the Putative Active Site in (−)-Pinoresinol-forming AtDIR6

The backbone RMSD between PsDRR206 and the published AtDIR6 homology model of Pickel *et al.* (11) is ∼5 Å, varying depending on which residues are superimposed. Three β-strands are out of register by two residues each with respect to their counterparts in the experimentally determined x-ray structure. Although the same side chains occupy the core of the β-barrel, they are misplaced because of incorrect alignment of neighboring strands. Our own models (5) were similarly poor. Such results are not unexpected given the low sequence similarity (∼17% identity in the core β-barrel) between AtDIR6 and AtAOC2, the template used to generate the homology model.

Nonetheless, the model of Pickel *et al.* (11) was sufficient to obtain a molecular replacement solution for the PsDRR206 crystal structure. This is because the core β-barrel of the template, AtAOC2 (PDB code 2BRJ) aligns very well with that of PsDRR206 (RMSD 3.2 Å over 113 residues). The structure has a RMSD 2.2 Å with *P. patens* AOC (PpAOC2, PDB code 4H69; Fig. 6*B*, which itself has a 1 Å RMSD alignment with AtAOC2). More specifically, because side chain atoms are not typically used in finding structure solutions by molecular replacement, the misalignment of several β-strands did not matter in this instance so long as positions of backbone atoms in the barrel are not perturbed by the misalignment.

**FIGURE 9.**
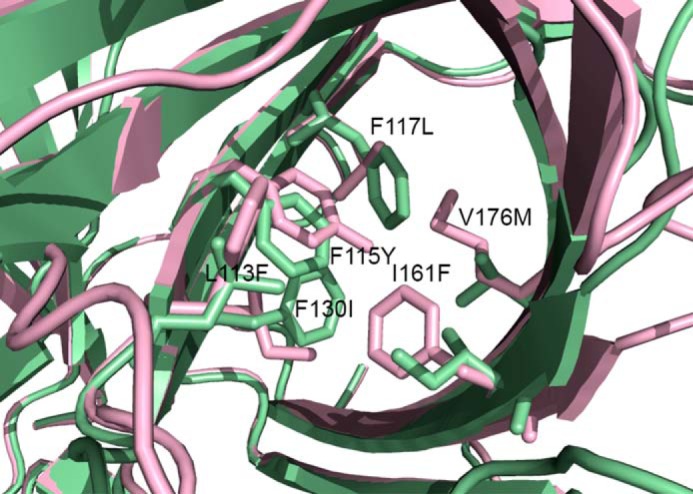
**AtDIR6 homology model (*lavender*) generated from the PsDRR206 x-ray structure (*green*) on which it is superimposed.** Differentially conserved active site side chains from (+)- and (−)-pinoresinol-forming DPs are shown and labeled in terms of the PsDRR206 residue type and number then the equivalent AtDIR6 residue type. (Valine 176 is substituted with threonine in the (+)-pinoresinol-forming DP ScDIR (5).)

**FIGURE 10.**
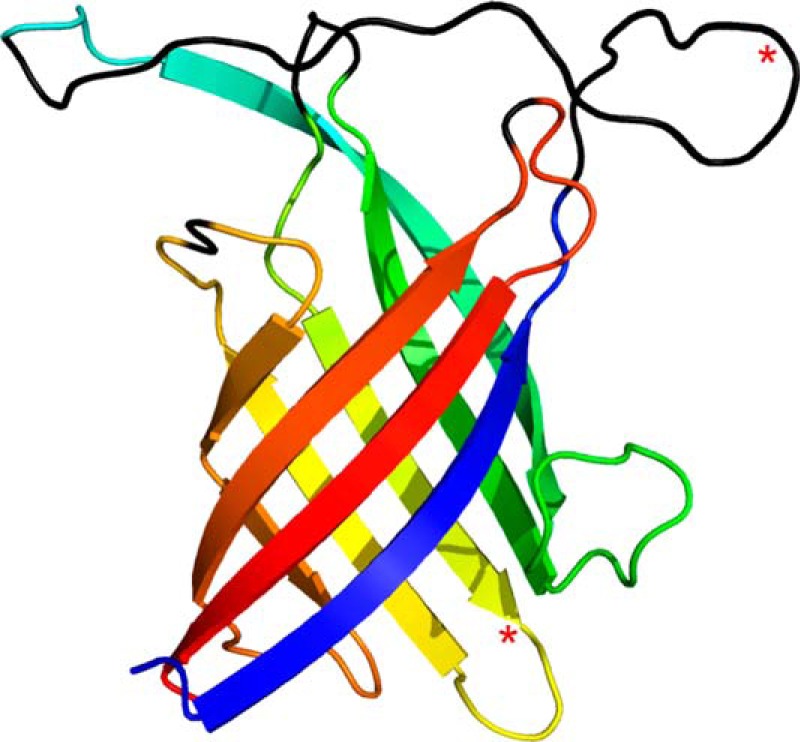
**PsDRR206 structure with residues in missing loops built in as disordered polypeptide chains (*black*) and subjected to backbone torsion minimization.** Putative *N*-glycosylation sites (Asn^50^ and Asn^120^) are indicated with *red asterisks*.

Using the PsDRR206 x-ray structure as a template, a new homology model of AtDIR6 was thus constructed to investigate the basis for differing stereoselectivities in pinoresinol-forming DPs. (Fig. 9). Five of the six residues that are differentially conserved among (+)- and (−)-pinoresinol-forming DPs from a range of plant species cluster together in the deep interior of the putative active site pocket. A seventh active site residue (Val^176^ in PsDRR206), not included in the original set because it is substituted with Thr in one (+)-pinoresinol-forming DP, becomes Met in (−)-pinoresinol-forming DPs. These residues are located opposite each another on either side of the pocket, in the center of one or the other of the two highly curved antiparallel β-sheets that form the β-barrel. Other active site residues nearer the mouth of the pocket are strictly conserved, such as Asp^40^, Arg^141^, and Thr^163^. We propose that the two different sets of differentially conserved residues force CA^•^ to bind such that only the *si* (for (+)-pinoresinol (**2a**)) or the *re* (for (−)-pinoresinol (**2b**)) face is exposed and available to couple with a second CA^•^ (or possibly CA), which itself must be oriented properly, perhaps by residues in the β1-β2 loop. Uncovering specific details of the mechanism, such as the actual roles of Asp^40^ and Arg^141^ and the orientation of each substrate, will likely require structural characterization of DPs with substrate or product analogues. Missing residues in loops surrounding the putative active site at the top of the β-barrel were also modeled to establish the range through which their motion can occur assuming the core barrel structure remains intact (Fig. 10). In particular, the 23 missing residues in the β1-β2 loop span a distance of ∼32 Å, conceivably providing ample potential to adopt a range of conformations.

##### Mechanistic Considerations

The trimeric DP structure shows that each active site is isolated from the others and will not allow two substrates bound at two different active sites to interact. In light of this finding, the most likely mechanism is that a single active site binds two substrates that combine to form product. The putative PsDRR206 binding pocket would thus bind two CA^•^ in an orientation allowing *si-si* coupling to form the (+)-pinoresinol antipode (**2a**), whereas the homologue AtDIR6 would afford the corresponding (−)-enantiomer (**2b**) through *re-re* coupling. However, a variation in which one CA^•^ and one coniferyl alcohol (**1**) bind and react cannot be ruled out.

Docking studies (Fig. 7) suggested that two CA^•^ substrates could bind in the active site, as could an 8–8′ *bis*-quinone methide (Fig. 1*A*). Although two positions of the methoxy group (*syn* or *anti*) with respect to C8 are possible in the quinone methide, discrimination of *si* and *re* faces that determine the stereochemistry at C8/8′ in pinoresinol (**2**) does not require the methoxy group to be in one position or the other. After the *bis*-quinone methide is formed, each of the 9/9′ -OH groups could nucleophilically attack the C7/C7′ quinone methide moieties to form the pinoresinol furanofuran product. Both cyclizations apparently occur stereospecifically, with no rotation about C7-C8, because this would instead lead to 7-epi-pinoresinol (or *bis* 7-epi-pinoresinol if a 180° rotation about both C7-C8 bonds occurred). This suggests that the *bis*-quinone methide intermediate is not released from the active site and that both substrates are bound such that both 8–8′ coupling and C7-O9′ and C7′-O9 ring closure occur in the bound state.

Pinoresinol (**2**) formation probably concludes with protonation of the quinone oxygen after rearomatization to form a phenol. Residue Asp^40^ in the putative binding pocket could be the source of this proton. Alternatively, if CA^•^ is bound in the opposite lengthwise orientation, the positively charged side chain of Arg^141^ could stabilize the quinone form of the phenolic oxygen in CA^•^, helping to localize the radical at C8 and favor coupling there. Indeed, binding in either orientation potentially provides ample opportunity for π-stacking interactions with aromatic residues in the active site (Fig. 7*B*). Asp^40^ and Arg^141^ are conserved in all DPs, whereas AOC does not have the corresponding residues, suggesting that they are required for substrate binding and activity specific to DPs, rather than for structural stability.

The 2-fold rotational symmetry of the pinoresinol molecule may have helped shape previous hypotheses (10, 11) for the mechanism of its formation involving a DP dimer with a single CA^•^ substrate bound to each monomer. The structure presented here is not consistent with such a mechanism, without invocation of a substrate-induced head to head joining of two trimers, for which there is currently no evidence.

Interestingly, in addition to stereoselective control over monolignol coupling in lignan biosynthesis, another study has also reported DP domain involvement in lignification in the root Casparian strip (39). Although this is in conceptual agreement with our earlier considerations of DPs and proteins harboring dirigent (substrate binding) sites/domains for lignan biosynthesis and lignification (8, 40), respectively, it must be emphasized that most DPs still await discovery of their true physiological functions, as well as in terms of chemical structural and *in vitro* function analyses. In due course, we will obtain definitive mechanistic models for substrate binding, orientation, coupling, intramolecular cyclization, and product release.

## Footnotes

3 D. S. Dalisay, K. W. Kim, C. Lee, H. Yang, O. Rübel, B. P. Bowen, L. B. Davin, and N. G. Lewis, submitted for publication.

## Footnotes

2 The abbreviations used are:

DP: dirigent protein
AOC: allene oxide cyclase
CA^•^: coniferyl alcohol derived free radical
PDB: Protein Data Bank
RMSD: root mean square deviation.
